# Towards Recyclable NAD(P)H Regeneration Catalysts

**DOI:** 10.3390/molecules17089835

**Published:** 2012-08-15

**Authors:** Miriam de Torres, Jonas Dimroth, Isabel W. C. E. Arends, Juliane Keilitz, Frank Hollmann

**Affiliations:** 1 Departamento de Química Orgánica, Universidad de Zaragoza, Pedro Cerbuna 12, E-50009, Spain; 2 Department of Chemistry, Technische Universiät Berlin, Straße des 17, Juni 124, Berlin 10623, Germany; 3 Department of Biotechnology, Delft University of Technology, Julianalaan 136, Delft 2628BL, The Netherlands; Email: i.w.c.e.arends@tudelft.nl; 4 Freie Universität Berlin, Institute of Chemistry and Biochemistry, Takustraße 3, Berlin 14195, Germany

**Keywords:** biocatalysis, cofactor regeneration, chemoenzymatic cascades, immobilized catalysts

## Abstract

Rh(III)-TsDPEN, an immobilized analog of the well-known [Cp*Rh(bpy)(H_2_O)]^2+^ was evaluated as a heterogeneous, recyclable regeneration catalyst for reduced oxidoreductase cofactors [NAD(P)H]. Repeated use of this catalyst was established and the catalytic properties were initially investigated. Apparently, Rh(III)-TsDPEN is prone to severe diffusion limitations, necessitating further developments. Overall, a promising concept for chemoenzymatic redox catalysis is proposed, which may overcome some of the current limitations such as catalyst cost and incompatibility of Rh with some biocatalysts.

## 1. Introduction

Pentamethylcyclopentadienyl rhodium-complexes ([Cp*Rh(L^⌒^L)]^n+^, wherein L^⌒^L is a bidentate ligand) have received considerable interest as non-enzymatic regeneration catalysts for reduced nicotinamide cofactors and flavin cofactors [1,2,3,4,5,6,7,8,9,10,11]. In particular their very broad activity spectrum with respect to temperature and pH makes them interesting, broadly applicable alternatives to the commonly used enzymatic systems [12].

**Scheme 1:**
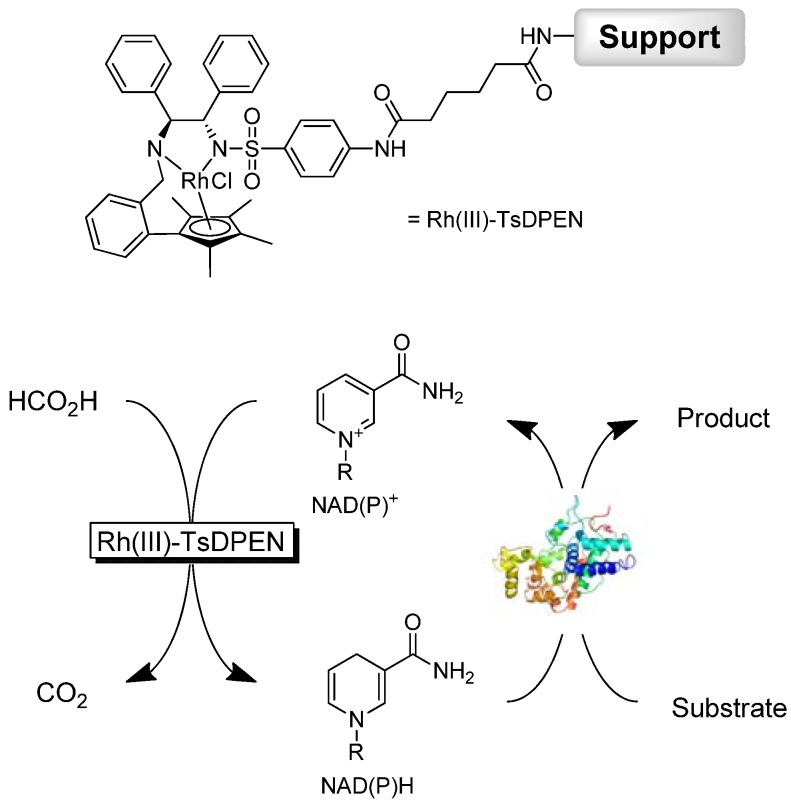
Immobilized Cp*Rh complex (Rh(III)-TsDPEN) evaluated in this study; and its application to promote NAD(P)H-regeneration. Support: Surface-functionalized poly(ethylene) sinter chips [17].

Some challenges, however, still have to be met *en route* to practical applicability of this regeneration concept. Particularly, recycling of the precious Rh-catalyst has so far been seldom addressed. Also, the mutual inactivation of regeneration- and biocatalyst [13,14,15] calls for a solution to achieve robust chemoenzymatic reactions schemes. Recently, Lütz and coworkers reported on a polymer-modified version of the well-known [Cp*Rh(bpy)(H_2_O)]^2+^[14]. Here, mass-enlargement of the transition metal catalyst enabled spatial separation from the enzyme by membranes and thereby prevented the above-mentioned inactivation. Inspired by these works we became interested in the recently described tethered Rh(III)-TsDPEN complex (Scheme 1). This heterogenized version of the piano-stool Cp*Rh complex has been described as efficient and recyclable transfer hydrogenation catalyst [16,17] Therefore, we evaluated Rh(III)-TsDPEN as recyclable alternative to the commonly used low-molecular weight [Cp*Rh(bpy)(H_2_O)]^2+^ complexes.

## 2. Results and Discussion

**Figure 1:**
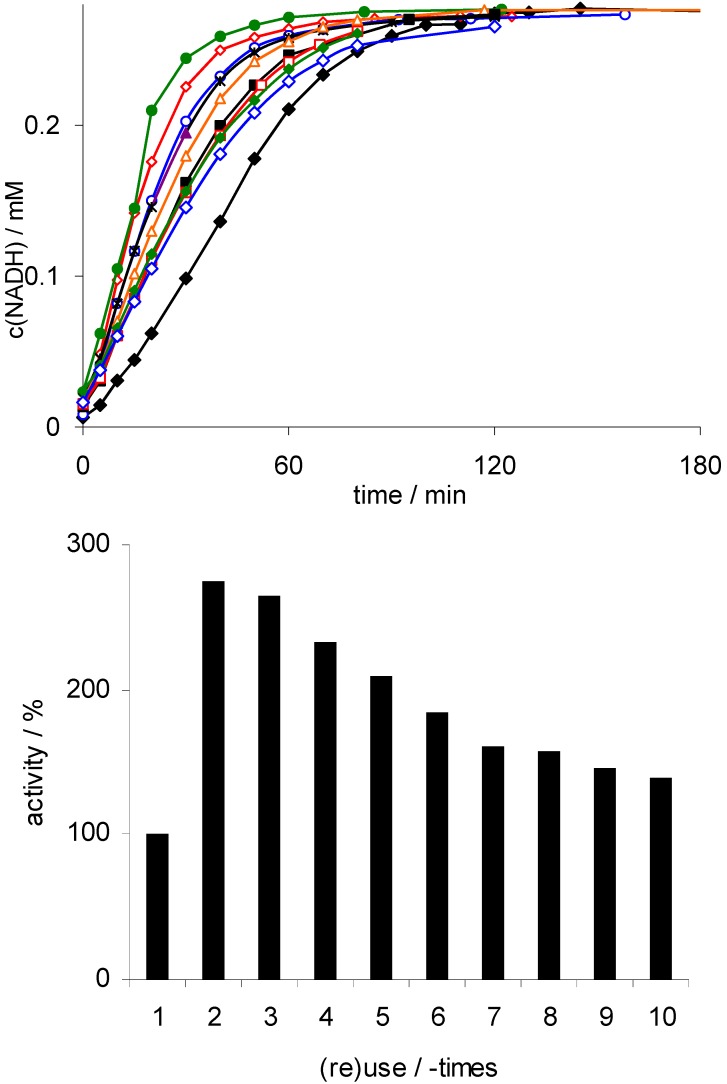
Recycling experiments with Rh(III)-TsDPEN. Upper: time courses of the single experiments (1: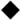; 2: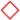; 3: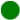; 4: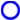; 5: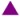; 6: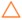; 7: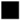; 8: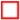; 9: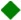; 10: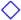); lower: initial rates. Conditions: 50 mg Rh(III)-TsDPEN (0.35 μmol), c(NAD^+^)_0_ = 0.25 mM, 50 mM phosphate buffer pH 7, T = 30 °C, c(NaHCO_2_) = 150 mM, 1,000 rpm; 100% corresponds to an NADH-generation rate of 0.21 mM·h^−1^.

Indeed, incubation of Rh(III)-TsDPEN with either NAD^+^ or NADP^+^ in the presence of formate (as reductant) lead to the formation of the enzymatically active reduced NAD(P)H (as demonstrated by UV/Vis spectroscopy and by activity assays with NADH-dependent dehydrogenases, data not shown). Encouraged by these results, we further investigated the catalytic properties of Rh(III)-TsDPEN with respect to activity and stability. Figure 1 shows the results of a first recycling experiment performed under arbitrarily chosen conditions.

Interestingly, an almost 3-fold activation of the catalyst was observed after the first use, which was followed by a gradual decrease during the successive experiments converging to approx 140% of the initial activity in the tenth use. Currently, we can only speculate about the nature of these observations. The initial activation may be rationalized by a displacement of the Cl^−^ counter ion in the Rh ligand sphere by water and/or phosphate. This should facilitate coordination of formate to the Rh central atom to form the catalytically active hydrido complex. Possibly then, the Rh-central atom is slowly poisoned by buffer impurities. Principally also leaching of the metal catalyst from the solid support could occur, however at least under storage conditions, no leaching was detectable. Further investigations will be necessary to fully understand the apparent ‘deactivation’ and to exclude leaching under reaction conditions.

It is also interesting to note that the catalytic activity of Rh(III)-TsDPEN was some 40% higher at pH 6. The decrease of both the initial activation and the subsequent activity, were less pronounced than at pH 7 (Supporting Information). However, similar effects have been reported for Rh(III)-TsDPEN in asymmetric transfer hydrogenation reactions (ATH) under acidic conditions [16].

It should be mentioned here that compared to the commonly used [Cp*Rh(bpy)(H_2_O)]^2+^ performance reported previously, the activity of Rh(III)-TsDPEN fell back approximately one order of magnitude (TOF of 2.5 h^−1^ as compared to approximately 36 h^−1^ under comparable conditions, respectively). To a large extent this is due to the difference in the bidentate ligand used. Using Cp*Rh complexed with a (biotinylated) aminosulfonamide ligand also lead to a reduction in NAD^+^-reduction to approximately 4 h^−1^ (as determined for the soluble catalyst) [18]. Hence, further optimization of the ligand system may lead to significant improvements in the catalyst activity. Nevertheless, also compared to the soluble aminosulfonamide-complexed catalyst, a significantly lower rate was observed for which we suspected diffusion limitation to be responsible. In fact, the shaking rate of the reaction vessel had a significant influence on the overall rate (supporting information).

**Figure 2:**
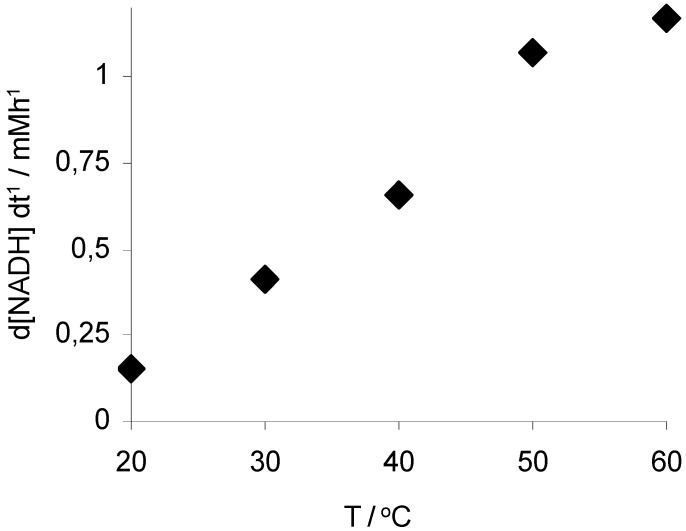
Temperature-dependence of the NAD^+^-reduction. Conditions: 50 mg Rh(III)-TsDPEN (0.35 μmol), c(NAD^+^)0 = 0.25 mM, c(NaHCO_2_) = 150 mM, 1,000 rpm.

This hypothesis is also supported by the, at first unexpected, activity profile of Rh(III)-TsDPEN with temperature (Figure 2). In contrast to our previous experience [12] the NAD^+^ reduction rate correlated only linearly with temperature. This apparent deviation from exponential behavior may be rationalized assuming diffusion of the substrates (HCO_2_^−^ and/or NAD(P)^+^, respectively) to be overall rate limiting. According to the Stokes-Einstein equation [19] the diffusion coefficient correlates linearly with temperature. An abnormal correlation of activity and temperature (>50 °C) was also observed using supported Rh(III)-TsDPEN as an ATH-catalyst. The authors hypothesized CO formation and subsequent catalyst inhibition to account for this observation [16]. However, further experiments will be necessary to clarify this issue.

**Figure 3:**
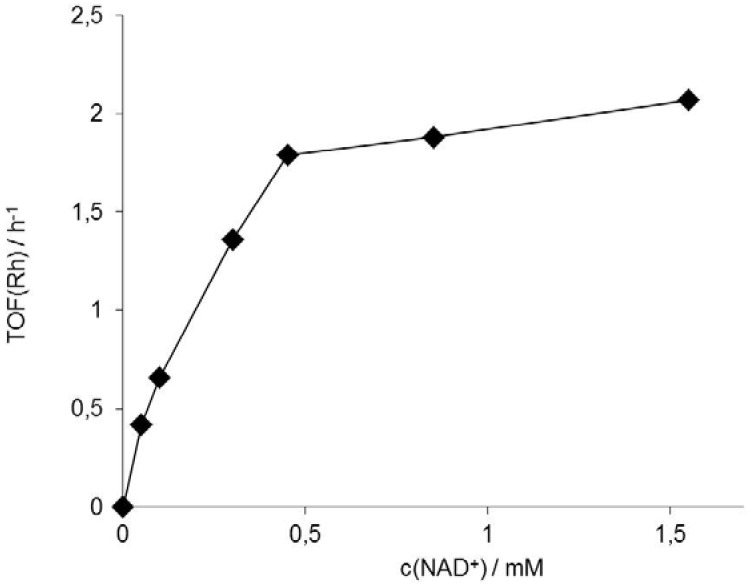
Dependence of Rh(III)-TsDPEN activity on c(NAD^+^)_0_, expressed as TOF(Rh). Conditions: 50 mg Rh(III)-TsDPEN (0.35 μmol), T = 30 °C, c(NaHCO_2_) = 150 mM, 1,000 rpm.

Therefore, we investigated the kinetic parameters of the Rh(III)-TsDPEN-driven reduction of NAD^+^ in some more detail. Due to the coordination equilibrium of both formate and NAD^+^ to the Rh-central atom, a Michelis-Menten-type behavior of Rh(III)-TsDPEN activity on c(NAD^+^) and c(HCO_2_^−^) can be expected [2]. In fact, this saturation type behavior was found also in case of Rh(III)-TsDPEN (Figure 3). While the apparent K_M_ value for formate (approximately 10 mM) was comparable to previously reported values, K_M_(NAD^+^) was significantly increased to approximately 250 μM (as compared to only 9 μM for [Cp*Rh(bpy)(H_2_O)]^2+^) [12]. Obviously, heterogenization affects the low-molecular weight formate ion to a lesser extent than the relatively large NAD^+^. V_max_ estimated from these experiments was around 2.5 h^−1^ (expressed as Rh turnover frequency). As mentioned above, this is one order of magnitude lower than determined for the soluble [Cp*Rh(bpy)(H_2_O)]^2+^ complex. It remains to be investigated whether this is due to the difference in the chelating ligand, the reduced flexibility of the Cp* ligand or results from the heterogeneous nature of Rh(III)-TsDPEN.

## 3. Experimental

Chemicals. Rh(III)-TsDPEN was prepared as previously described [17]. All other chemicals were purchased from Sigma-Aldrich (Zwijndrecht, Netherlands) in the highest purity available and used as received.

Recycling experiments were performed on 2 mL scale using 50 mg of Rh(III)-TsDPEN suspended in buffer (potassium phosphate, 50 mM, pH 7) at 30 °C. The reaction mixture was supplemented with 150 mM NaHCO_2_ and 0.25 mM NAD^+^ (final concentrations) and shaken at 1,000 rpm. At intervals, samples were withdrawn and analyzed spectrophotometrically (340 nm, ε = 6.22 mM^−1^cm^−1^). After each reaction, the catalyst was filtered off, washed with distilled water and either reused immediately or after storage at room temperature.

Kinetic measurements were performed as described above only varying the parameters (concentrations, temperature) as indicated in the figure captions. 

## 4. Conclusions

Overall, we have demonstrated the application of an immobilized, recyclable Rh-catalyst for the regeneration of reduced nicotinamide cofactors. Admittedly, the catalyst reported here is not practical to promote NAD(P)H-dependent redox reactions on a preparative scale. Mainly its catalytic performance is somewhat lower than that of comparable soluble counterparts and this severely impairs its applicability. However, the results presented here strongly support the assumption of diffusion (especially of the large nicotinamide cofactors) being overall rate limiting. Hence, this study lays the basis for further catalyst improvements, e.g., supports with higher porosity and for alternative ligand systems. Nevertheless, it was demonstrated that the immobilized Rh catalyst can be reused at least 10 times. Further studies to fully understand the factors influencing Rh(III)-TsDPEN-activity and to elucidate its potential in redox biocatalysis are currently underway.

## Supplementary Materials

Supplementary materials can be accessed at: http://www.mdpi.com/1420-3049/17/8/9835/s1.

## References

[1] Hollmann F., Arends I.W.C.E., Buehler K. (2010). Biocatalytic Redox Reactions for Organic Synthesis: Nonconventional Regeneration Methods. ChemCatChem.

[2] Lee S.H., Nam D.H., Kim J.H., Baeg J.-O., Park C.B. (2009). Eosin Y-Sensitized Artificial Photosynthesis by Highly Efficient Visible-Light-Driven Regeneration of Nicotinamide Cofactor. ChemBioChem.

[3] Song H.K., Lee S.H., Won K., Park J.H., Kim J.K., Lee H., Moon S.J., Kim D.K., Park C.B. (2008). Electrochemical regeneration of NADH enhanced by platinum nanoparticles. Angew. Chem. Int. Ed..

[4] Kim J.H., Lee S.H., Lee J.S., Lee M., Park C.B. (2011). Zn-containing porphyrin as a biomimetic light-harvesting molecule for biocatalyzed artificial photosynthesis. Chem. Commun..

[5] Kim J.H., Lee M., Lee J.S., Park C.B. (2012). Self-Assembled Light-Harvesting Peptide Nanotubes for Mimicking Natural Photosynthesis. Angew. Chem. Int. Ed..

[6] Kohlmann C., Greiner L., Leitner W., Wandrey C., Lütz S. (2009). Ionic Liquids as Performance Additives for Electroenzymatic Syntheses. Chem. Eur. J..

[7] Hildebrand F., Lütz S. (2007). Electroenzymatic synthesis of chiral alcohols in an aqueous-organic two-phase system. Tetrahedron: Asymmetry.

[8] de Gonzalo G., Ottolina G., Carrea G., Fraaije M.W. (2005). [Cp*Rh(bpy)(H_2_O)]^2+^ as a coenzyme substitute in enzymatic oxidations catalyzed by Baeyer-Villiger monooxygenases. Chem. Commun..

[9] Bernard J., van Heerden E., Arends I.W.C.E., Opperman D.J., Hollmann F. (2012). Chemoenzymatic reduction of conjugated C=C-bonds. ChemCatChem.

[10] Mifsud Grau M., Poizat M., Arends I.W.C.E., Hollmann F. (2010). Phosphite-driven regeneration of reduced enzyme cofactors. Appl. Organometal. Chem..

[11] Hollmann F., Schmid A. (2009). Towards [Cp*Rh(bpy)(H_2_O)]^2+^-promoted P450 catalysis: Direct regeneration of CytC. J. Inorg. Biochem..

[12] Hollmann F., Witholt B., Schmid A. (2002). [Cp*Rh(bpy)(H_2_O)]^2+^: A versatile tool for efficient and non-enzymatic regeneration of nicotinamide and flavin coenzymes. J. Mol. Catal. B Enzym..

[13] Poizat M., Arends I.W.C.E., Hollmann F. (2010). On the nature of mutual interaction between [Cp*Rh(bpy)(H_2_O)]^2+^ and enzymes—Analysis and potential remedies. J. Mol. Catal. B Enzym..

[14] Hildebrand F., Lütz S. (2009). Stable Electroenzymatic Processes by Catalyst Separation. Chem. Eur. J..

[15] Haquette P., Talbi B., Barilleau L., Madern N., Fosse C., Salmain M. (2011). Chemically engineered papain as artificial formate dehydrogenase for NAD(P)H regeneration. Org. Biomol. Chem..

[16] Dimroth J., Schedler U., Keilitz J., Haag R., Schomäcker R. (2011). New Polymer-Supported Catalysts for the Asymmetric Transfer Hydrogenation of Acetophenone in Water—Kinetic and Mechanistic Investigations. Adv. Synth. Catal..

[17] Dimroth J., Keilitz J., Schedler U., Schomäcker R., Haag R. (2010). Immobilization of a Modified Tethered Rhodium(III)-p-Toluenesulfonyl-1,2-diphenylethylenediamine Catalyst on Soluble and Solid Polymeric Supports and Successful Application to Asymmetric Transfer Hydrogenation of Ketones. Adv. Synth. Catal..

[18] Köhler V., Wilson Y.M., Dürrenberger M., Ghislieri D., Churakova E., Quinto T., Knörr L., Häussinger D., Hollmann F., Turner N.J. (2012). New Synthetic Cascades by Combining Biocatalysts with Artificial Metalloenzymes. Nat. Chem..

[19] Einstein A. (1905). The motion of elements suspended in static liquids as claimed in the molecular kinetic theory of heat. Ann. Phys.-Berlin.

