# Supplementary material for: Towards Recyclable NAD(P)H Regeneration Catalysts
Source: Molecules. 2012 Aug 15;17(8):9835–41. doi: 10.3390/molecules17089835 (PMC6268897; doi:10.3390/molecules17089835)

Recycling experiments for Rh(III)-TsDPEN at pH6

These experiments were performed analogous to the ones described in the manuscript. The only difference was the pH of the reaction mixture (pH 6 instead of 7).

Figure S1. Time courses of the NAD^+-reduction experiments using recycled Rh(III)-TsDPEN**.** Conditions: 50 mg Rh(III)-TsDPEN (0.35 µmol), $c(NAD^+)_0$ = 0.25 mM, 50 mM phosphate buffer pH 6, T = 30 °C, $c(NaHCO_2)$ = 150 mM; (1:◆; 2:◇, 3:●, 4:○; 5:▲; 6:△; 7:■; 8:□; 9:◆; 10:◇).

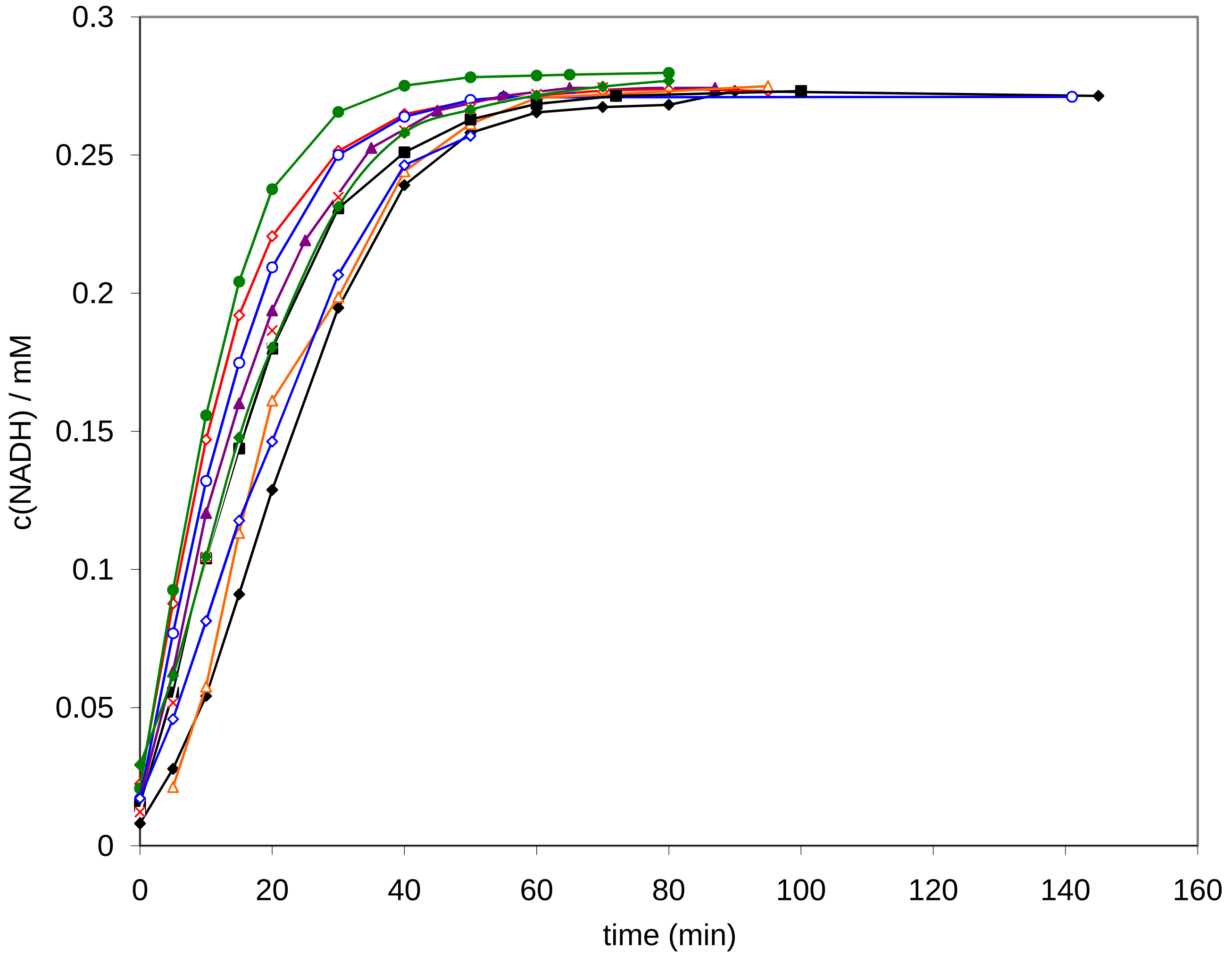

Figure S2. Relative initial rates of the recycling experiments performed at pH6. Conditions: 50 mg Rh(III)-TsDPEN (0.35 μmol), c(NAD^+)$_0$ = 0.25 mM, 50 mM phosphate buffer pH 6, T=30 °C, c($NaHCO_2$) = 150 mM.

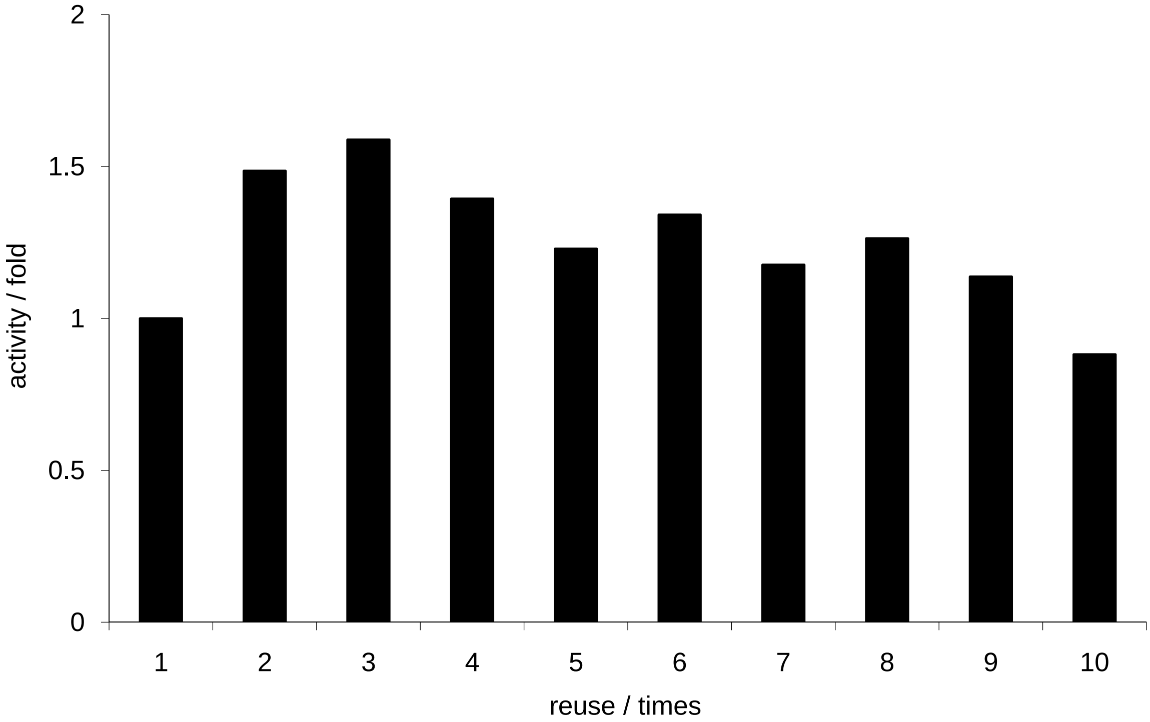

Influence of the Agitation Speed on the Rate of the Rh(III)-TsDPEN-Catalyzed Reduction of NAD$^+$

As shown in Figure S3, the agitation speed had a direct influence on the rate of the NADH regeneration rate of Rh(III)-TsDPEN.

Interestingly, there seemed to be an optimal agitation speed as the NADH formation rate at maximal agitation speeds was reproducibly somewhat lower than at e.g., 1,000 rpm. One possible explanation may be aerobic reoxidation of the intermediate hydridorhodium complex. However, further experiment would be necessary to substantiate this hypothesis.

Figure S3. Influence of the agitation speed on the NAD$^+$-reduction rate. Conditions: 50 mg Rh(III)-TsDPEN (0.35 μmol), c(NAD$^+$)$_0$ = 0.25 mM, 50 mM phosphate buffer pH 7, T = 30 °C, c($NaHCO_2$) = 150 mM.

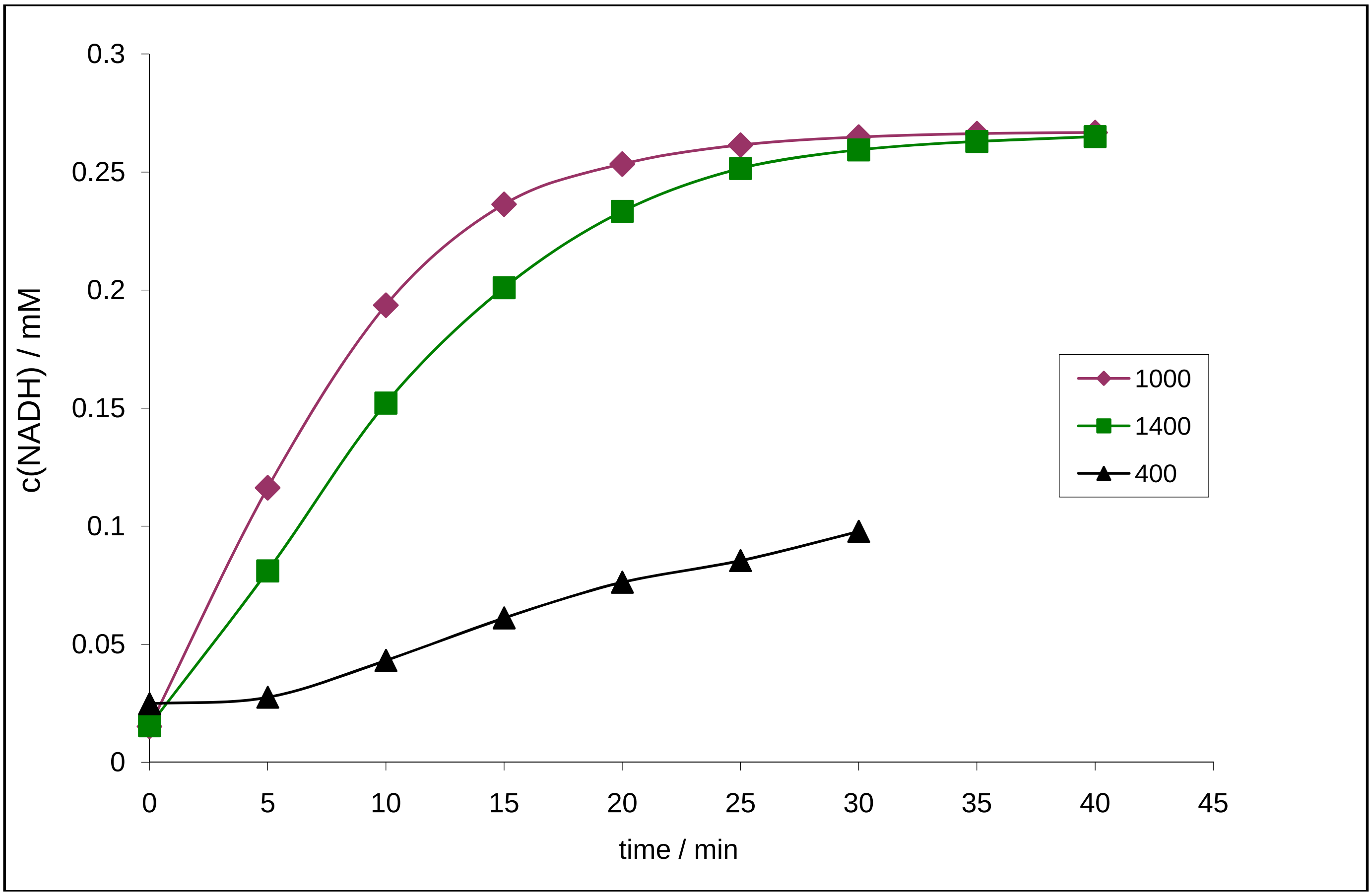

Influence of the formate concentration on the rate of the Rh(III)-TsDPEN-catalyzed reduction of NAD^+

Figure S4. Influence of the formate concentration on the activity of Rh(III)-TsDPEN (as expressed as turnover frequency, TOF). Conditions: 50 mg Rh(III)-TsDPEN (0.35 µmol), $c(NAD^+)_0$ = 0.25 mM, 50 mM phosphate buffer pH 7, T=30 °C.

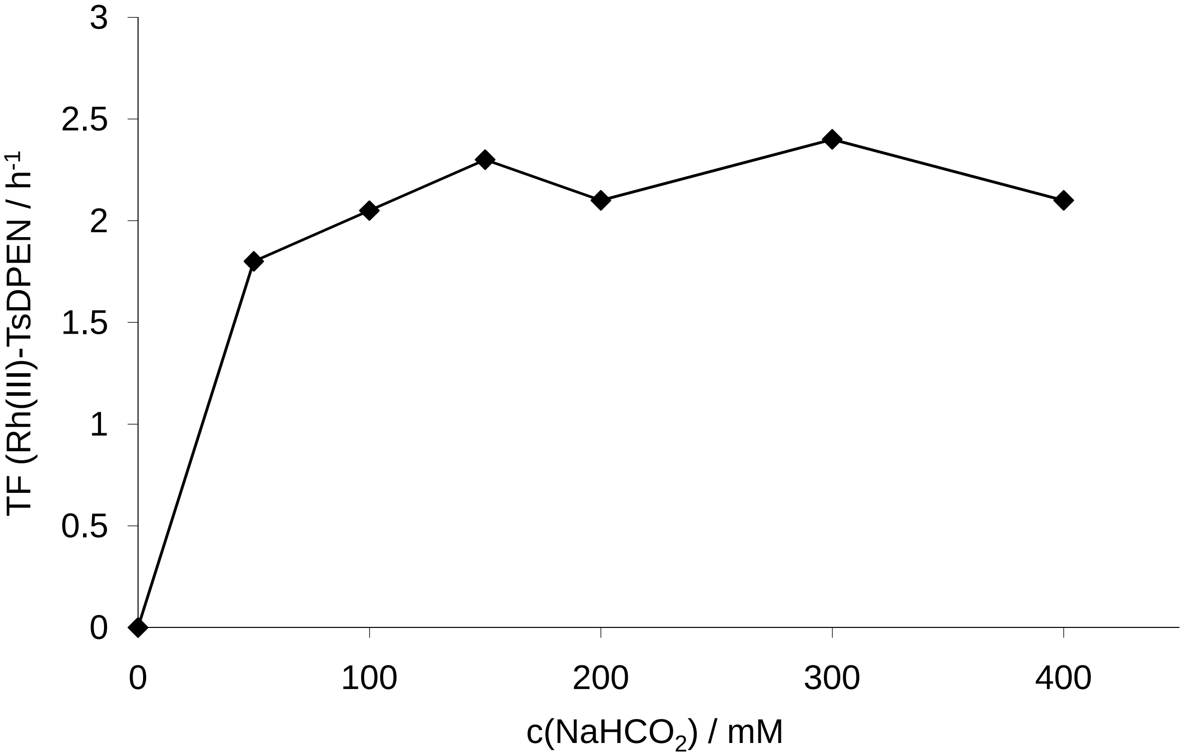

Supplement: Supplementary file 1 [file molecules-17-09835-s001.pdf]
